# Enantioselective Resolution of (*R*,*S*)-Carvedilol to (*S*)-(−)-Carvedilol by Biocatalysts

**DOI:** 10.1007/s13659-016-0118-2

**Published:** 2017-01-07

**Authors:** Swetha Ettireddy, Vijitha Chandupatla, Ciddi Veeresham

**Affiliations:** University College of Pharmaceutical Sciences, Kakatiya University, Warangal, Telangana 506 009 India

**Keywords:** Carvedilol, Whole-cell microorganisms, Triacylglycerol lipase, Immobilization, Stereoinversion

## Abstract

Among the microorganisms employed in the study, *Aspergillus niger* (GUFCC5443), *Escherichia coli* (ATCC9637), *Streptomyces halstedii* (CKM-2), *Pseudomonas putida* (NCIB9494), *Cunninghamella elegans* (NCIM689) and *Sphingomonas paucimobilis* (NCTC11030) were capable for the enantioselective conversion of racemic Carvedilol. Immobilization technique enhanced the enantioselectivity of microorganisms and thus increased the enantiomeric purity of the drug. Excellent enantiomeric ratios (E) were found in reactions catalyzed by immobilized *A. niger* and *E. coli* with values 174.44 and 104.26, respectively. Triacylglycerol lipase from *Aspergillus niger* was also employed in this study as a biocatalyst which resulted in the product with 83.35% enantiomeric excess (*ee*) and E of 11.34 while the enzyme on immobilization has yielded 99.08% ee and 216.39 E. The conversion yield (C%) of the drug by free-enzyme was 57.42%, which was enhanced by immobilization to 90.51%. Hence, our results suggest that immobilized triacylglycerol lipase from *A. niger* (Lipase AP6) could be an efficient biocatalyst for the enantioselective resolution of racemic Carvedilol to (*S*)-(−)-Carvedilol with high enantiomeric purity followed by immobilized cultures of *A. niger* and *E. coli*.

## Introduction

In a chiral drug, each enantiomer has its own particular pharmacological profile. There are several advantages for single enantiomer products when compared to their racemates like more selective and comparatively simple pharmacodynamic as well as pharmacokinetic profile, potential for an improved therapeutic index and very less probability for complex drug interactions [1]. Though there are quite a few ways to obtain chiral molecules in commercially pure form like resolution of racemates, chiral pool synthesis, laboratory-invented chiral building blocks, asymmetric synthesis and bio-transformations, no single method is ideal. Hence combination of biotransformation with chromatographic resolution [1] was employed in the present study. Development of a single enantiomer from a previously marketed racemate is known as chiral switch [2] and there are different new resolution procedures for obtaining pure enantiomer from a racemic mixture with 100% yield, theoretically, like dynamic kinetic resolution, stereoinversion by deracemization, cyclic racemization and enantioconvergent processes, etc. Among them stereoinversion facilitates in obtaining 100% of one pure enantiomer from its racemic mixture by inverting the other substrate enantiomer. The mechanism involves the transformation of one enantiomer of a racemate to an achiral intermediate which is further irreversibly transformed to the opposite enantiomer; thus the product contains one pure enantiomer at maximum. This process can be facilitated by using either redox catalysts, in pure or semipure form, or biocatalysts [3]. The complex architecture of microorganisms and isolated enzymes made them as efficient catalysts for enantioselective conversions [4]. Unlimited availability of enzyme, internal cycling of co-factors and vigorous speed of catalysis made the whole-cell microorganisms as efficient catalysts for enantioselective resolution of racemic drugs as well as racemic intermediates. For example, *R*-(+)-aminophosphonic acid is an HIV protease inhibitor, antibacterial and antifungal agent and is resolved from its racemate using fungi, *Cunninghamella echinulata*, with 42% enantiomeric excess (*ee*) [5]. But with whole-cell microorganisms there will be competition between enzymes for the substrate which might reduces the stereoselectivity [4]. This limitation can be overcome by using isolated enzymes, along with suitable co-factors [6]. Some examples of enzyme catalyzed chiral switches include Stiripentol to (*R*)-(+)-stiripentol (anti-convulsant drug) [7], Amphetamine to (*S*)-(+)-amphetamine (CNS stimulant) [8], Ibuprofen to (*S*)-(+)-ibuprofen (non-steroidal anti-inflammatory drug) [9], *α*-Lipoic acid to (*R*)-(+)-lipoic acid (anti-HIV and antitumor agent) [*10*], etc. Among different enzymes, lipases are uniquely stable in non-polar organic solvents [11] with increased catalytic activity and stereoselectivity. These are readily available from various microorganisms, do not require co-factors and accept wide range of non-natural substrates [12]. Hence lipases can be used for the resolution of various chiral compounds. For example, synthesis of long acting β_2_ receptor agonist (Salmeterol, Formoterol, Bambuterol, etc.) requires two chiral intermediates, (*R*)-1-(4-(benzyloxy)-3-nitrophenyl)-2-bromoethanol and (*R*)-*N*-(1-(4-methoxyphenyl) propan-2-yl) acetamide and are resolved from their corresponding racemates using Amano PS-30 lipase and lipase from *Candida antarctica*, respectively [13]. Lipases can catalyze various reactions like hydrolysis, aminolysis, esterification, transesterification, etc. and the means followed can be described as ping–pong bi–bi mechanism. Accordingly, first step involves nucleophilic attack on the carbonyl group of a drug which results in acyl-enzyme intermediate. This step is promoted by serine, histidine and aspartate residues (catalytic triad) of enzyme lipase. The acyl-enzyme intermediate then reacts with a nucleophile like water, alcohols or amines regenerating the enzyme along with the product [14]. The immobilization technique offers several advantages over the free-biocatalysts such as an increase in enzyme stability, longevity and catalytic activity [11], increase in stereoselectivity [15], possibility of repeated use of biocatalysts, easy cell separation from liquid medium and ease of handling. Immobilization of cells and enzymes can be done by various methods like adsorption (carrier-binding), cross-linking (covalent), entrapment and membrane confinement [16]. Among these methods, entrapment in calcium alginate beads is most widely used technique in pharmaceutical industry because alginate can form gels under mild conditions, and it is non-toxic, non-pathogenic, cheap and readily available [17]. (*R*)-(−)-mandelic acid, key intermediate in the production of cephalosporins, penicillins, antitumor agents, is resolved from racemic mandelonitrile using alginate-immobilized nitrilase [13].

Carvedilol is a non-selective, *β*-adrenergic receptor antagonist and *α*_1_-adrenoceptor blocker. As its structure possesses one chiral center, it exists in two enantiomeric forms [18]. The overall cardioprotective action of Carvedilol is attributed to its (*S*)-(−)-enantiomer and which is also less hepatotoxic than racemic mixture and (*R*)-(+)-enantiomer [19]. Till date there has been no report on the biotransformation of racemic Carvedilol to its (*S*)-(−)-enantiomer. Hence, this drug is a suitable candidate for demonstration of biocatalytic resolution to provide a single active enantiomer compound. Thus, the present study aimed for the enantioselective resolution of racemic Carvedilol to its (*S*)-(−)-enantiomer using various microorganisms like *Bacillus subtilis* [20]*, Escherichia coli* [21]*, Pseudomonas putida* [22]*, Sphingomonas paucimobilis* [23]*, Rhodococcus erythropolis* [24], *Streptomyces halstedii* [25]*, Aspergillus niger* [26], *Candida parapsilosis* [27]*, Geotrichum candidum* [28], *Rhizopus oryzae* [29], *Cunninghamella elegans* [30], *Cunninghamella blakesleeana* [31] and an enzyme, triacylglycerol lipase from *Aspergillus niger* (Lipase AP6) [32], which were reported earlier for their ability to convert racemates to the eutomers (active enantiomer), enantioselectively.

## Results and Discussion

**Fig. 1:**
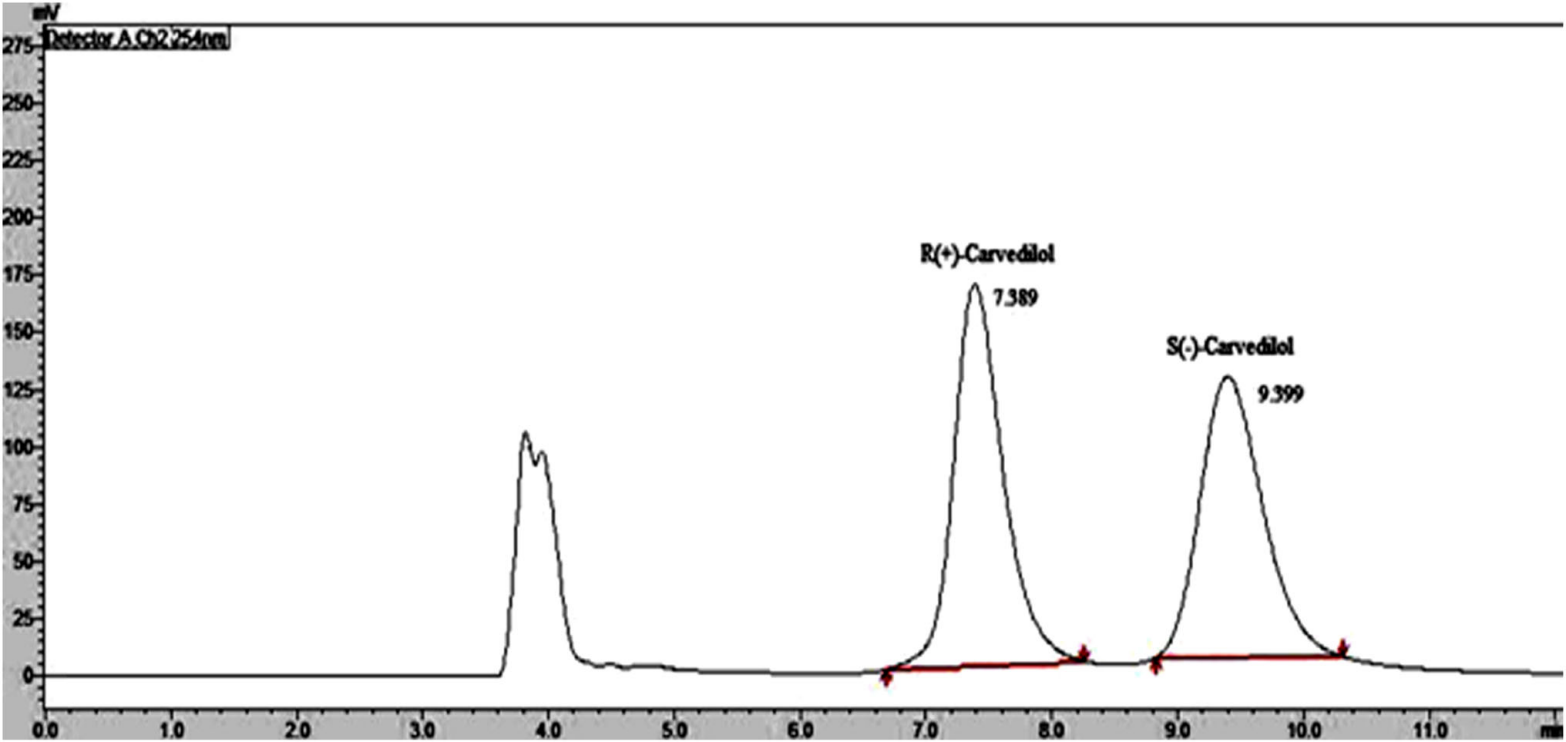
Chromatogram of racemic Carvedilol (1 µg/mL) [R(+)-Carvedilol and S(−)-Carvedilol]

In this study the enantioselective resolution was confirmed by estimating the enantiomeric purity of the product by using an earlier reported chiral HPLC method [33]. The racemic Carvedilol was analyzed by the chiral HPLC method and retention time of the (*R*)-(+)- and (*S*)-(−)-enantiomers were found as 7.389 and 9.399 min, respectively (Fig. 1).

### Whole-Cell and Immobilized-Microorganisms Catalyzed Enantioselective Resolution of Carvedilol

**Fig. 2:**
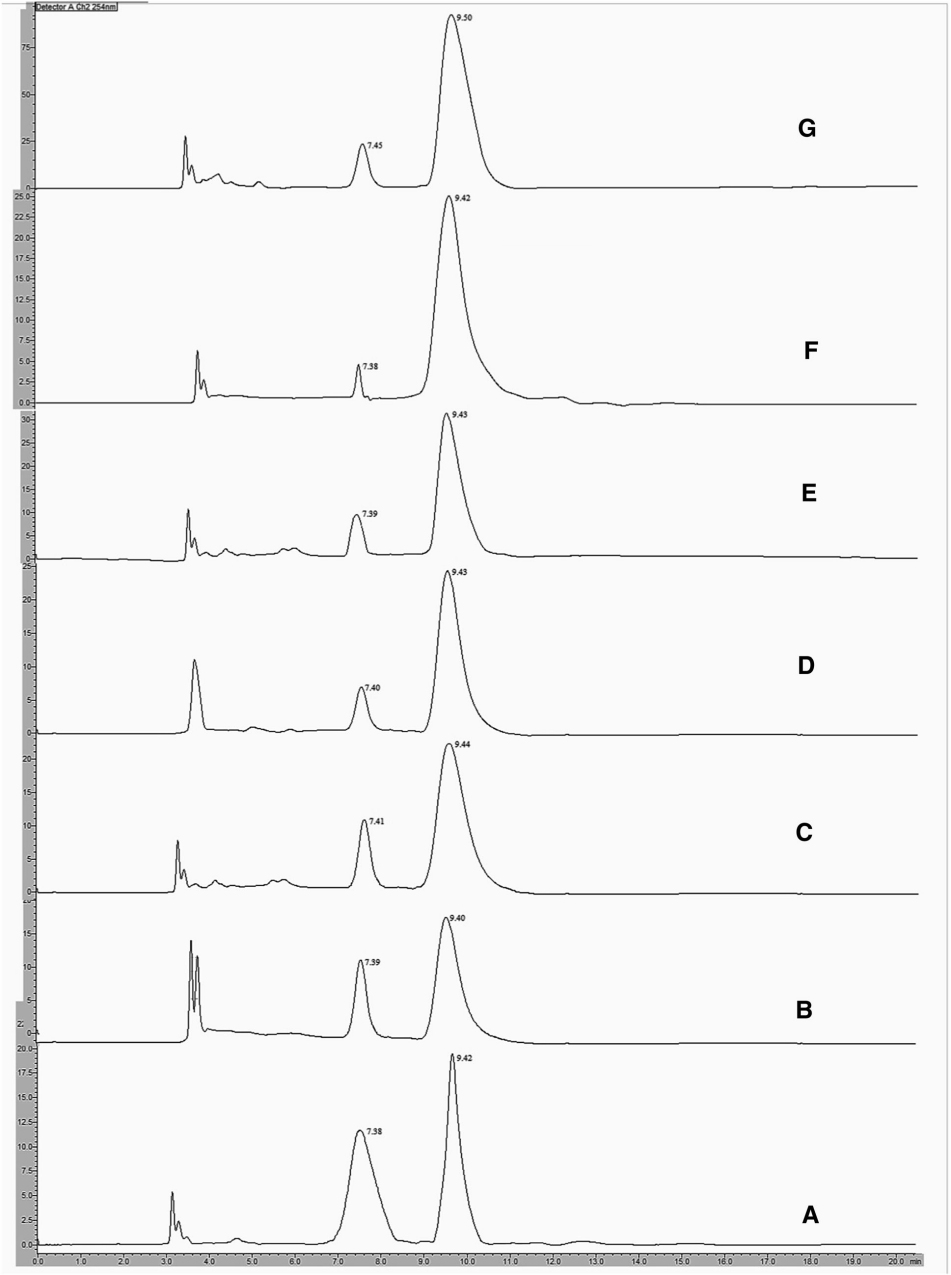
Chromatograms of Carvedilol after incubation with whole-cell cultures and free-enzyme. **a** *Sphingomonas paucimobilis*; **b** *C. elegans*; **c** *P. putida*; **d** *Streptomyces halstedii*; **e** *E. coli*; **f** *A. niger*; **g** Lipase AP6 enzyme

**Fig. 3:**
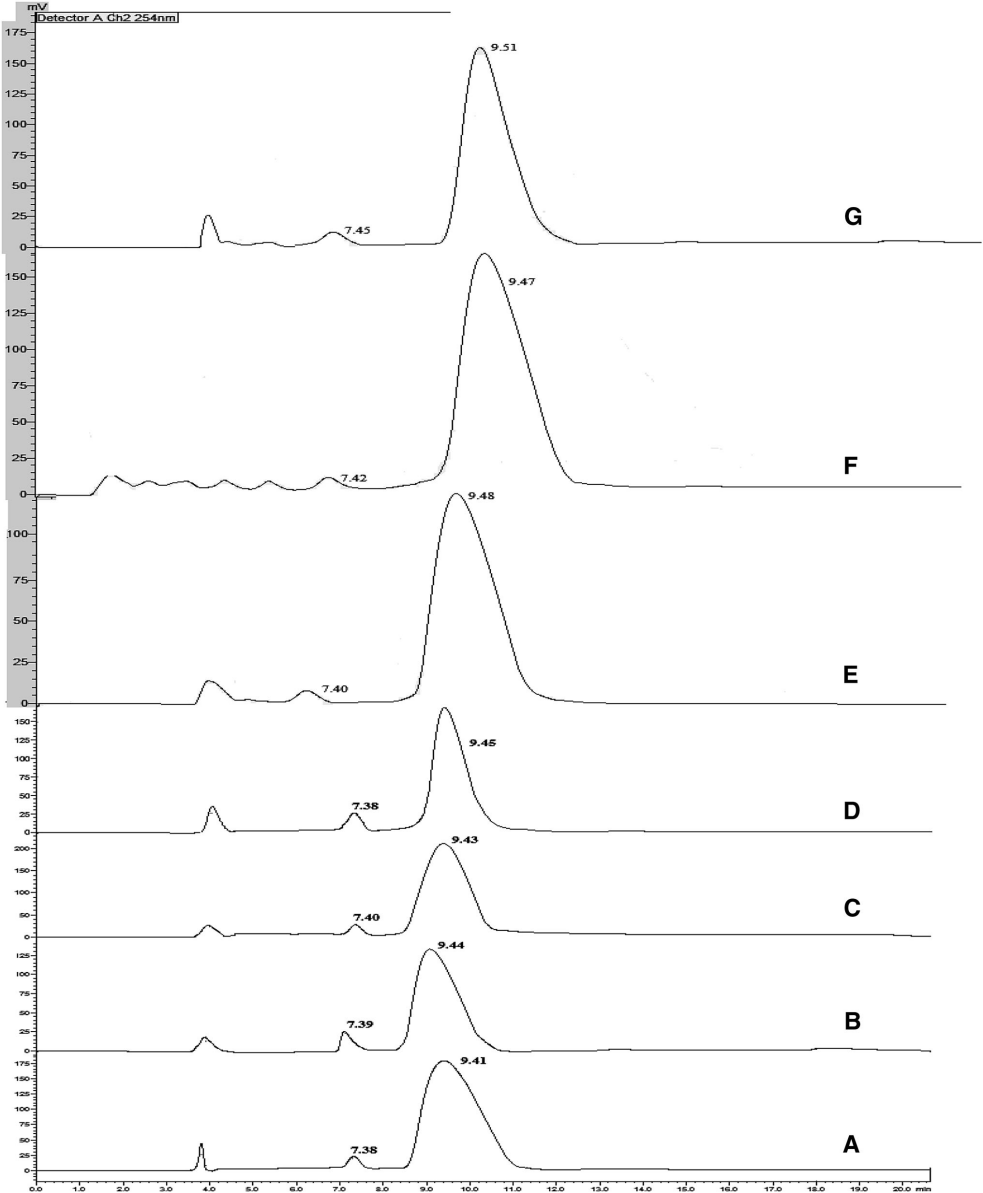
Chromatograms of Carvedilol after incubation with immobilized-cell cultures and immobilized-enzyme. **a** *Sphingomonas paucimobilis*; **b** *C. elegans*; **c** *P. putida*; **d** *Streptomyces halstedii*; **e** *E. coli*; **f** *A. niger*; **g** Lipase AP6 enzyme

Among the employed microbial cultures only *A. niger*, *E. coli*, *Streptomyces halstedii*, *P. putida*, *C. elegans* and *Sphingomonas paucimobilis* were capable for the enantioselective resolution of racemic Carvedilol. The enantioselective conversion was gradually increased up to tenth day of incubation and after that there was a decrease in concentration of drug with no conversion. Hence the maximum enantiomeric excess was seen in tenth day samples. The chromatograms of Carvedilol after incubation with whole-cell *Sphingomonas paucimobilis*, *C. elegans*, *P. putida*, *Streptomyces halstedii*, *E. coli* and *A. niger* cultures for 10 days were shown in Fig. 2a–f, respectively. The enantiomeric purity of the drug present in the tenth day samples, collected from whole-cell and immobilized microorganism catalyzed studies, was assessed by calculating the enantiomeric excess (*ee*), enantiomeric ratio (E) and conversion yield (C%). The whole-cell cultures were shown to have ability for the enantioselective conversion of Carvedilol with moderate to good ee% and the values were shown in Table 1. The enantiomeric purity of the drug (product) obtained by the reaction catalyzed by whole-cell *A. niger* culture was high, followed by the reactions catalyzed by *E. coli*, *Streptomyces halstedii*, *P. putida*, *C. elegans* and *Sphingomonas paucimobilis*. But E values of the reactions catalyzed by these cultures, except *A. niger*, were worst to poor [34] and the values were shown in Table 1. These results indicate that the whole-cell microbial cultures has very less enantioselectivity and hence are practically not acceptable. Therefore the cultures were immobilized to improve their enantioselectivity [11], which will enhance the enantiomeric purity of the drug [35]. Immobilization of the cultures has not only improved their enantioselectivity but also simplified the sample collection and facilitated the efficient extraction of the drug from these samples. Comparative enantioselective conversion of Carvedilol by whole-cell and immobilized cell cultures was shown in Table 1. From the results it was clear that the immobilization has improved the enantioselectivity of all these microbial cultures. The E values of the reactions catalyzed by immobilized cultures of *Sphingomonas paucimobilis* and *C. elegans* were enhanced from worst to moderate, which results in good enantiomeric excess of the drug, comparatively. The E value of the reaction catalyzed by immobilized *Streptomyces halstedii* has improved from poor to good and hence the enantiomeric purity of the product was good (high enantiomeric excess). The enantioselectivities of *E. coli* and *A. niger* were also improved by immobilization and their E values were found to be excellent as they were greater than 100 [34]. The improved enantioselectivities of these two cultures lead to very high enantiomeric purity of the product. The chromatograms of Carvedilol after incubation with immobilized-cell *Sphingomonas paucimobilis*, *C. elegans*, *P. putida*, *Streptomyces halstedii*, *E. coli* and *A. niger* cultures for 10 days were shown in Fig. 3a–f, respectively.

**Table 1:** Enantioselective conversion of Carvedilol after incubation with the whole-cell as well as immobilized-cell cultures for 10 days

S. no	Microorganism	Whole-cell incubation			Immobilized-cell incubation		
		ee (%)	E	C (%)	ee (%)	E	C (%)
1	*Aspergillus niger*	82.13	10.19	87.95	98.86	174.44	66.31
2	*Escherichia coli*	76.07	7.36	14.52	98.10	104.26	44.06
3	*Streptomyces halstedii*	67.00	5.06	32.03	95.59	44.35	64.63
4	*Pseudomonas putida*	60.15	4.02	46.16	94.67	36.52	34.92
5	*Cunninghamella elegans*	57.51	3.71	85.11	89.10	17.34	78.21
6	*Sphingomonas paucimobilis*	53.21	3.27	94.03	87.24	14.67	72.39

Overall, enantioselectivities of the whole-cell cultures, which were responded for the enantioselective conversion of Carvedilol, were improved by immobilization and lead to enhanced enantiomeric purity of the product. But, among all the cultures *A. niger* and *E. coli* were yielded very high enantiomeric excesses with excellent enantioselectivites after immobilization.

### Free- and Immobilized-Enzyme Catalyzed Enantioselective Resolution of Carvedilol

The samples collected at specific time intervals from free- and immobilized enzyme catalyzed studies were analyzed by chiral HPLC method for enantioselective resolution of racemic Carvedilol and their comparative enantiomeric excesses were shown in Table 2. The ee% of Carvedilol was gradually increased by both free- and immobilized-enzyme up to 20th h and a slight increase was seen at 24th h. There wasn’t any improvement observed in *ee* on incubation for further longer periods. Hence enantiomeric purity of the drug in 24th h sample was assessed by calculating *ee*, E and C%. As the enzyme immobilization improves the catalytic activity [11], stability and enantioselectivity of the enzyme, it enhanced the enantiomeric purity of the drug when compared to its free form. The enantioselectivity of the enzyme was enhanced by immobilization and this was confirmed by enhanced E value (216.39) of the reaction catalyzed by enzyme after immobilization when compared to that of the reaction catalyzed by free-enzyme (E = 11.24). The conversion yield of the drug was also enhanced (C% = 90.51) by reaction catalyzed by immobilized-enzyme when compared to that of the reaction catalyzed by free-enzyme (C% = 57.42). From Table 2, it was clear that the enantiomeric purity (*ee*) of the drug in the samples collected from immobilized-enzyme catalyzed study was high at each time point when compared to that of the samples collected from free-enzyme catalyzed study. Chromatograms of Carvedilol after incubation with free- and immobilized-Lipase AP6 enzyme for 24 h were shown in Figs. 2g and 3g, respectively.

**Table 2:** Enantiomeric purity of Carvedilol after incubation with free- and immobilized-enzyme

S. no.	Time interval (h)	Enantiomeric excess (*ee*)	
		Free-enzyme	Immobilized-enzyme
1	4	5.93	7.52
2	8	12.35	14.90
3	12	28.87	41.56
4	16	53.56	69.74
5	20	82.94	99.02
6	24	83.66	99.08

On the whole, excellent enantiomeric purity of the drug was obtained by the immobilized cultures and enzyme when compared to their respective free forms. These results were in accordance with the results obtained by Suzuki et al. [36], and according to them calcium alginate immobilized-cell cultures of *Alcaligenes species* or *Pseudomonas species* are capable for the production of (*R*)-3-chloro-1,2-propanediol (intermediate in synthesis of chiral synthons) from its racemate with high yield, high chemical and optical purity when compared to their whole-cell cultures. The results also strengthen the conclusion made by Wang et al. [37], i.e., immobilization technique will improve the enantioselectivity and operational stability of biocatalyst. When compared to immobilized *A. niger*, immobilized lipase was found to be more enantioselective and thus responsible for excellent enantiomeric purity of the Carvedilol. Hence, the immobilized-lipase was found to be more suitable biocatalyst for enantioselective resolution of racemic Carvedilol with excellent enantiomeric purity followed by immobilized *A. niger* and *E. coli.*

## Experimental Section

### General Experimental Procedures

Incubation was done in refrigerated shaker incubator of model Innova 4230, New Brunswick Scientific Co., Inc., (NJ, USA). Sample analysis was done by using a chiral column purchased from Phenomenex (USA) (Lux Cellulose-4; 250 × 4.6 mm; 5 µ particle size) and an Ultra Fast Liquid Chromatograph of Shimadzu (Kyoto, Japan) equipped with binary pump (LC 20AD), UV/Visible detector (LC 20A) and Rheodyne injector port. Lab solutions software was employed for the HPLC analysis.

Racemic Carvedilol was kindly gifted by Symed labs limited, Medak (India). (*S*)-(−)-Carvedilol and (*R*)-(+)-Carvedilol were purchased from the Toronto Pharmaceuticals (Canada). Calcium chloride, sodium alginate, sodium chloride and di-potassium hydrogen phosphate were purchased from Hi Media Laboratories Pvt. Ltd., Mumbai (India). Solvents (*n*-heptane, isopropanol and methanol) of HPLC grade were purchased from Sigma Aldrich Chemicals Pvt. Ltd., Maharashtra (India). Laboratory grade ethyl acetate was purchased from Finar limited, Ahmadabad, Gujarat (India).

### Cultures and Growth Media

The lyophilized cultures of *B. subtilis*, *E. coli*, *P. putida*, *Sphingomonas paucimobilis*, *R. erythropolis*, *Streptomyces halstedii*, *A. niger*, *C. parapsilosis*, *G. candidum*, *R. oryzae*, *C. blakesleeana*, and *C. elegans* were purchased from Microbial Type Culture Collection and Gene Bank (MTCC) (Chandigarh, India)/National Collection of Industrial Microorganisms (NCIM) (NCL, Pune, India). Suitable growth media and triacylglycerol lipase from *A. niger* (Lipase AP6) were purchased from Hi Media Laboratories Pvt. Ltd., Mumbai (India). All the lyophilized cultures were rejuvenated and incubated in refrigerated shaker incubator under MTCC/NCIM specified growth conditions. Sub-culturing onto solid and liquid media in regular intervals was continued for maintaining the cultures, which were stored in refrigerator at 4 °C.

### Incubation Protocol

In biotransformation experiments a two-stage fermentation protocol will enhances the metabolic activities of microorganisms and different types of liquid growth media have been used for the selected microbial cultures. *E. coli*, *B. subtilis*, *P. putida* and *Sphingomonas paucimobilis* were cultured using Nutrient broth as liquid growth medium and its composition includes Beef extract (1 g/L), Yeast extract (2 g/mL), peptone (5 g/L), sodium chloride (5 g/L) and distilled water (up to 1 L). Final pH of the medium was adjusted to 7.2. *R. oryzae*, *C. elegans* and *C. blakesleeana* were cultured by using Potato Dextrose broth medium and its composition includes Potato (scrubbed and diced, 200 g/L), Dextrose (20 g/L) and distilled water (up to 1 L). *C. parapsilosis*, and *G. candidum* were cultured by using Malt Yeast medium and its broth was prepared by dissolving Malt extract (3 g/L), Yeast extract (3 g/L), Peptone (5 g/L), Glucose (10 g/L) in distilled water (up to 1 L) and the pH was adjusted to 6.2. *R. erythropolis* and *Streptomyces halstedii* were cultured by using Streptomyces medium and composition of the broth medium includes Glucose (4 g/L), Yeast extract (4 g/L), Malt extract (10 g/L), Calcium carbonate (2 g/L) and distilled water (up to 1 L). The pH of the medium was adjusted to 7.2. *A. niger* was cultured by using Malt extract broth medium, which was prepared by dissolving Malt extract (20 g/L) in distilled water (up to 1 L) and its pH was adjusted to 6.5.

In the present study, incubation protocol was followed according to the reported method [38], with few modifications. Briefly, first stage culture was initiated by inoculating 25 mL of sterile liquid medium with a loopful of freshly grown culture, under laminar airflow cabinet. These cultures were incubated at 120 rpm under MTCC/NCIM specified growth conditions for particular microorganism. Second stage culture was initiated by inoculating 25 mL of fresh sterile liquid media with 5–10% v/v inoculum from the first stage culture. Sterile liquid media used for fungal cultures was supplemented with 0.02% TritonX100 in order to get good dispersion of fungi in the media [39].

### Whole-Cell Microorganisms Catalyzed Enantioselective Resolution of Carvedilol

This biotransformation method was done according to the reported method [40], with few modifications. Briefly, the second stage suspension cultures (25 mL) of all experimental organisms were added with racemic Carvedilol solution in methanol (0.01 mg/mL culture). Prior to addition, the drug solution was filter sterilized using sterile syringe driven PVDF hydrophilic membrane filter (pore size-0.22 µm). The flasks were gently shaken immediately after the addition of drug for its even distribution. Each (culture + drug) incubation study was done in triplicate (n = 3) with suitable controls. In culture controls the second stage suspension cultures were added with 100 µL of methanol instead of the drug solution while the drug controls composed of sterile growth medium added with the drug solution. All the test and controls were incubated under specified conditions for 20 days, samples were collected (third, fifth, tenth, fifteenth and twentieth day), extracted and analyzed by chiral HPLC.

### Immobilized Microorganisms Catalyzed Enantioselective Resolution of Carvedilol

The microbial cultures which were responded for the enantioselective resolution were selected for this study. As the maximum extent of enantioselective conversion was seen up to tenth day, the incubation was carried for only up to 10 days. Whole cell microorganisms were immobilized by alginate-entrapment method described in Arpana et al. [41], with slight modifications. Briefly, second stage suspension cultures were filtered and the cells were washed with di-potassium hydrogen phosphate buffer (pH 6.8) followed by distilled water. These cells were added to 3% (w/v) of sodium alginate solution (1:1) with vigorous stirring to ensure even distribution. Using a syringe, the suspension was dropped into aqueous solution of calcium chloride (0.2 M) from a height of 15 cm to ensure the bead size as 2–3 mm. Resulted calcium alginate beads were allowed to stand in calcium chloride solution (1 h) for hardening and then they were washed with distilled water and used for the study. Immobilized cells in buffer (0.5% K_2_HPO_4_ and 0.5% sodium chloride) without drug considered as culture control while the drug in buffer without immobilized cells was considered as drug control. Except culture control all the flasks were added with racemic Carvedilol solution in methanol (0.01 mg/mL culture) and incubated under specific growth conditions for 10 days at 120 rpm. The study was performed in triplicate (n = 3). The samples collected were extracted and analyzed by chiral HPLC.

### Extraction and Sample Preparation

The samples collected were extracted with three volumes of ethyl acetate by vertex mixing for 1 min and the organic phase was collected. This process was repeated for three times and the combined organic phase was evaporated and dried in vacuum oven [40]. The residues were reconstituted with 1 mL methanol (HPLC grade), filtered through sterile filters (pore size-0.22 µm) and were analyzed by chiral HPLC.

### Free-Enzyme Catalyzed Enantioselective Resolution of Carvedilol

The reaction mixture was composed of isopropanol and *n*-heptane (6:4) along with racemic Carvedilol of concentration 2.5 mg/mL in methanol. The reaction was started by adding lipase (10 mg/mL) to the reaction mixture [12, 35] and was incubated at 120 rpm, at 25 °C for 36 h. Samples were collected (4th, 8th, 12th, 16th, 20th, 24th, 28th, 32nd, 36th h) and evaporated at room temperature. The residues were dissolved in methanol (HPLC grade), filtered through membrane filter (pore size-0.22 µm) and analyzed by chiral HPLC.

### Immobilized Enzyme Catalyzed Enantioselective Resolution of Carvedilol

Enzyme immobilization was done by reported method [41] with few modifications. Briefly, enzyme (10 g/L) was suspended in sterile phosphate buffer (pH 6.8) and was mixed with equal volume of sodium alginate solution (3% w/v). Using a syringe, this suspension was extruded into calcium chloride solution (0.2 M) from a height of 15 cm to yield calcium alginate beads (2 mm) and kept aside (1 h) for hardening. Then they were washed thrice with distilled water and employed for the study by adding them into reaction mixture along with racemic Carvedilol (2.5 mg/mL methanol). Reaction mixture with enzyme-alginate beads and without drug was considered as blank while the drug control consists of reaction mixture with the drug and without beads. All the test and controls were incubated at 120 rpm at 25 °C for 36 h, samples were collected (4th, 8th, 12th, 16th, 20th, 24th, 28th, 32nd, 36th h) and evaporated at room temperature. The residues were dissolved in methanol (HPLC grade), filtered through membrane filter (pore size-0.22 µm) and analyzed by chiral HPLC.

### HPLC Analysis

The samples prepared after extraction were analyzed by an earlier reported chiral HPLC method for the enantioselective resolution of racemic Carvedilol [33]. Accordingly, the stationary phase used was Phenomenex Lux Cellulose-4 column (250 × 4.6 mm; 5 µ particle size). Isopropanol and n-Heptane in 60:40 ratio had served as mobile phase while the flow rate was set to 1 mL/min. Sample injection volume was 20 µL, cell temperature was 40 °C and the detection wavelength used in the method was 254 nm.

For a resolution process to be valuable it must have high selectivity and which can be determined by estimating the enantiomeric ratio (E) of the process. E can be calculated by formula;

$$E = \frac{{1 + {\text{ee}_{\rm p}}}}{{1 - {\text{ee}_{\rm p}}}}$$

where ‘*ee*_p_’ is the enantiomeric excess of the product [35].

Generally in biocatalytic reactions, the E value is considered as worst if it is in between 1 and 5, poor if in between 5 and 10, moderate if in between 10 and 20 and as good if in between 20 and 100. If E value is above 100 then is considered as excellent [34]. If the reaction to yield high enantiomeric purity (high enantiomeric excess), then it should exhibit excellent enantioselectivity. For example, ee_P_ values yielded by the reactions with E values of approximately 19, 40 and 100 are 90, 95 and 98%, respectively. If E value is above 100 then it yields excellent enantiomeric purity (*ee* > 99%). The enantiomeric excesses of the substrate and the product (*ee*_S_ and *ee*_P_) of a reaction can be calculated using the following equations [42];

$$\% {\text{ee}_{\rm s}} = \frac{{{\text{R}} - {\text{S}}}}{{{\text{R}} + {\text{S}}}} \times 100$$

$$\% {\text{ee}_{\rm p}} = \frac{{{\text{R}} - {\text{S}}}}{{{\text{R}} + {\text{S}}}} \times 100$$

where ‘S’ and ‘R’ represent the chromatographic peak areas of the (*S*)- and (*R*)-enantiomers, respectively.

The conversion (C) yield can be calculated by the formula [43];

$${\text{C}} = 1 - \frac{{\left[ {\text{A}} \right] + [{\text{B}}]}}{{\left[ {\text{A}} \right]_{^\circ } + [{\text{B}}]_{^\circ } }}$$

where [A] and [B] are peak areas of (*R*)- and (*S*)-enantiomers respectively while [A]_°_ and [B]_°_, are the initial peak areas of (*R*)- and (*S*)-enantiomers, respectively [44]. The conversion yield is being expressed in %.

Enantiomeric excess, enantiomeric ratio and conversion yield of the reaction were calculated for every sample by using the formulae [35, 42, 43].
